# ESCAIDE 2018 – call for late breakers

**DOI:** 10.2807/1560-7917.ES.2018.23.36.1809062

**Published:** 2018-09-06

**Authors:** Eurosurveillance editorial team

**Affiliations:** 1 European Centre for Disease Prevention and Control (ECDC), Stockholm, Sweden

The 2018 European Scientific Conference on Applied Infectious Disease Epidemiology (ESCAIDE) will take place in Saint Julian's, Malta, between 21 and 23 November 2018.

A so called ‘Late Breaker’ session will be organised at ESCAIDE. The aim of this session is to give speakers an opportunity to give oral presentations of important new findings of recently conducted studies which could have immediate implications for public health.

The call for late breaker abstracts is open now and will close on 14 September 2018.

Please visit the conference website to read more about the eligibility criteria for abstract submission: https://www.escaide.eu/en/presenters/call-abstracts-2018.

